# Ferulic Acid: A Comprehensive Review

**DOI:** 10.7759/cureus.68063

**Published:** 2024-08-28

**Authors:** Jaganathan R Purushothaman, Md. Rizwanullah

**Affiliations:** 1 Department of Orthopedics, Saveetha Medical College and Hospital, Saveetha Institute of Medical and Technical Sciences (SIMATS) Saveetha University, Chennai, IND; 2 Center for Global Health Research, Saveetha Medical College and Hospital, Saveetha Institute of Medical and Technical Sciences (SIMATS) Saveetha University, Chennai, IND

**Keywords:** antioxidant activity, bioavailability, nanoparticles, solubility, ferulic acid

## Abstract

Ferulic acid (FA), a phenolic compound abundant in the cell walls of seeds, leaves, and roots of various fruits, vegetables, cereals, and grains, is renowned for its wide range of biological activities, including antioxidant, anti-inflammatory, antimicrobial, and anticancer properties. Despite its therapeutic potential, the clinical application of FA is hindered by challenges such as poor water solubility, limited bioavailability, rapid metabolism, and instability under physiological conditions. To address these issues, nanotechnology has emerged as a transformative approach, enhancing FA's pharmacokinetic profile. Various nanoparticle-based systems, including polymer-based and lipid-based nanoparticles, have been developed to encapsulate FA. These systems have demonstrated significant improvements in FA's solubility, stability, and bioavailability, with studies showing enhanced antioxidant activity and controlled release profiles. Further, the surface engineering of these nanoparticles provides targeted drug/phytochemical delivery potential. The targeted delivery of drugs/phytochemicals significantly enhances the therapeutic efficacy and minimizes systemic side effects. This review explores the therapeutic potential of FA, the limitations in its clinical application, and the advancements in nanoparticle-based delivery systems that are paving the way for its effective therapeutic use.

## Introduction and background

Ferulic acid (FA) is a ubiquitous phenolic compound extensively found in the plant kingdom, particularly in the cell walls of seeds, leaves, and roots of various fruits, vegetables, cereals, and grains [1]. FA has garnered substantial attention in recent decades due to its impressive spectrum of biological activities. As an integral component of various fruits, vegetables, and grains, FA is known for its potent antioxidant, anti-inflammatory, antimicrobial, and anticancer properties [2,3]. These attributes position it as a promising candidate for therapeutic applications, particularly in chronic diseases where oxidative stress and inflammation are key pathogenic factors. Despite its significant medicinal potential, the clinical translation of FA faces several formidable challenges, including poor solubility, limited bioavailability, and rapid metabolism [4]. Consequently, innovative drug delivery systems are being actively explored to enhance the therapeutic efficacy of FA.

Despite the excellent therapeutic effects, the complex physicochemical characteristics of FA significantly limit its clinical application. FA exhibits poor water solubility (<1 μg/mL) and a poor intrinsic dissolution rate, which hinders its absorption from the intestine and results in poor bioavailability. Further, FA undergoes rapid metabolism in the physiological environment and is excreted rapidly from the body. These factors necessitate a higher dose of FA to achieve therapeutic outcomes. Moreover, the stability of FA under physiological conditions is a concern, as it is prone to degradation in the presence of light, heat, and alkaline pH. These limitations underscore the need for advanced drug delivery systems that can improve FA's solubility, stability, and bioavailability [5,6].

In the last three decades, nanotechnology has emerged as a powerful tool to address these challenges, offering innovative solutions for the delivery of FA. Nanoparticles (NPs) have shown promise in enhancing the pharmacokinetic profile of FA. NPs enhance the therapeutic efficacy of FA by addressing several key limitations associated with its conventional delivery. NPs improve FA's solubility by encapsulating it in a matrix that is more compatible with physiological fluids, allowing for better absorption. NPs also protect FA from premature degradation and metabolism, ensuring that a higher concentration of the active compound reaches the systemic circulation. Additionally, NPs can be engineered to provide controlled and sustained release of FA, maintaining therapeutic levels in the bloodstream for longer periods. This reduces the frequency of dosing and improves patient compliance. Moreover, NPs can be designed to target specific tissues or cells, such as cancerous tissues or inflamed areas, by exploiting the enhanced permeability and retention effect or by incorporating ligands that bind to receptors overexpressed on target cells. This targeted delivery increases the concentration of the encapsulated drug at the desired site, enhancing its therapeutic effect and minimizing exposure to healthy tissues, thereby reducing potential side effects [7]. Through these mechanisms, NPs significantly improve the overall therapeutic potential of FA in treating various diseases. Among the various nanotechnology-based approaches, polymer-based NPs and lipid-based NPs have been extensively studied for the delivery of FA. These systems not only enhance the stability and solubility of FA but also allow for targeted delivery, minimizing systemic side effects and enhancing therapeutic effects. Additionally, NPs offer unique advantages in terms of biocompatibility, controlled release, and ease of administration [8,9].

Polymer-based NPs, such as chitosan, poly(lactic-co-glycolic acid) (PLGA), and polyethylene glycol (PEG) NPs, offer the advantage of biocompatibility and controlled release properties [10,11]. These NPs can encapsulate FA, protecting it from degradation and allowing for sustained release at the target site. The use of surface modifications, such as the attachment of targeting ligands, can further enhance the specificity of FA delivery to diseased tissues, minimizing systemic side effects [12]. Lipid-based NPs, including solid lipid nanoparticles (SLNs), nanostructured lipid carriers (NLCs), and liposomes, have also shown great promise in the delivery of FA. These NPs can improve the solubility and bioavailability of FA by encapsulating it within the lipid matrix [13,14]. Moreover, lipid-based NPs offer the advantage of biocompatibility and the ability to cross different biological barriers and show significant potential to achieve better therapeutic efficacy against various diseases [15].

This manuscript presents a comprehensive review of the pharmacological properties of FA, its biopharmaceutical challenges, and the innovative nanotechnology-based drug delivery systems designed to enhance its therapeutic delivery, providing a unique perspective on overcoming the clinical limitations of FA.

## Review

Overview, physicochemical characteristics, and pharmacological properties

**Figure 1:**
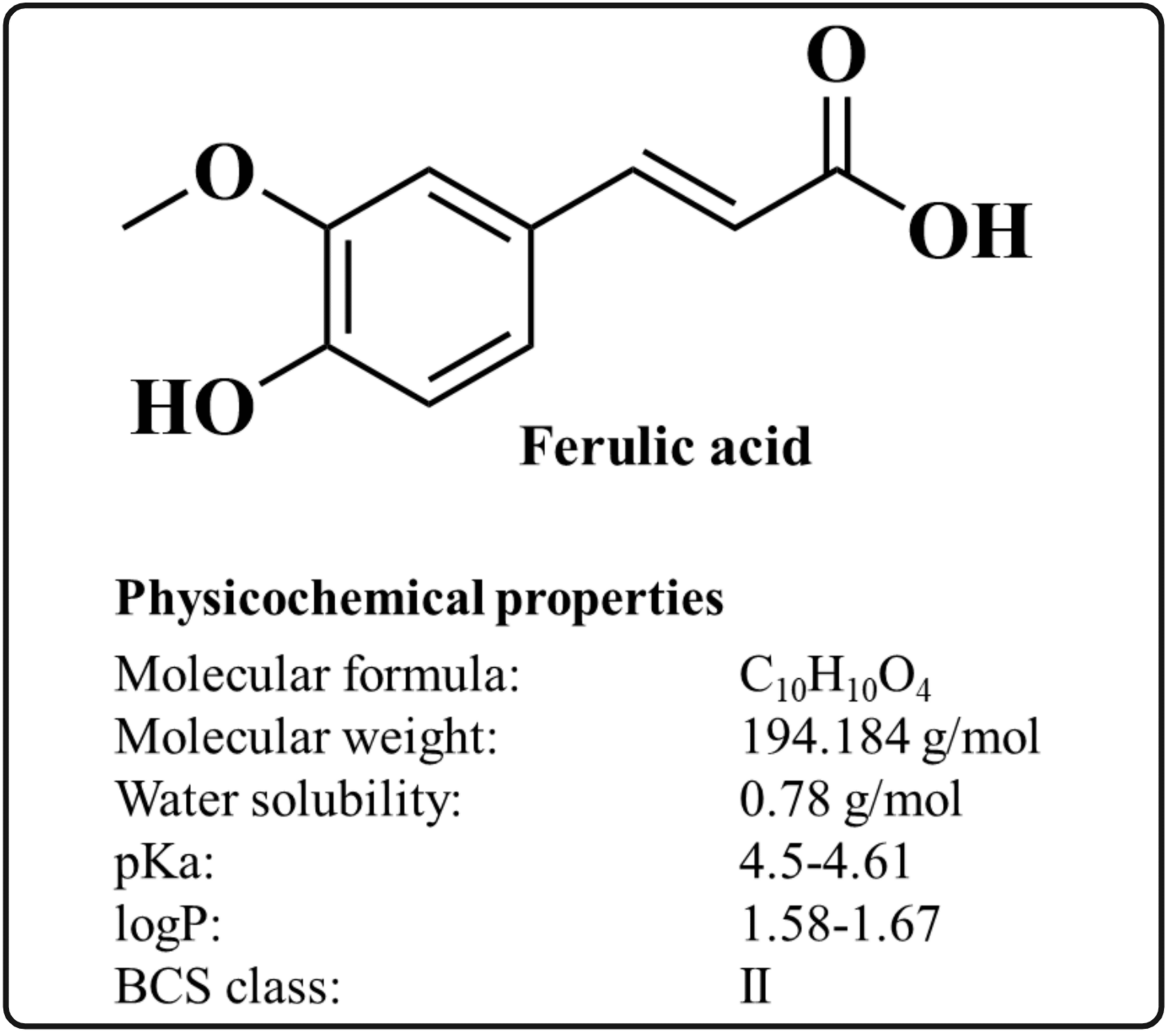
The chemical structure and physicochemical properties of FA. Image credit: Md. Rizwanullah

FA (4-hydroxy-3-methoxycinnamic acid) is a caffeic acid derivative widely found in vegetables and fruits [16,17]. The chemical structure of FA and its physicochemical properties are represented in Figure 1. Moreover, FA is also found in Chinese medicinal plants such as *Angelica sinensis*, *Cimicifuga racemosa*, and *Ligusticum chuangxiong* [18]. FA is a crystalline substance with a molecular formula of C_10_H_10_O_4_ and a molecular weight of 194.18 g/mol. It melts at temperatures between 168 and 172 °C [19]. FA remains stable below 76% relative humidity and under typical ambient conditions [20]. FA is freely soluble in organic solvents such as dimethyl sulfoxide, ethanol, methanol, propylene glycol, ethylene glycol, and isopropyl alcohol but insoluble in water [21,22]. FA belongs to the Class II drug according to the Biopharmaceutical Classification System [23]. When ingested orally, FA undergoes enterohepatic recycling and rapid metabolism through phase I enzymes such as cytochrome P450 monooxygenases, including CYP2D6 and CYP2C19 [24,25]. Further, FA and its esters display a safe plasma profile [26]. The pharmacological properties of FA are discussed below.

Antioxidant Activity

The antioxidant activity of FA is primarily attributed to its unique chemical structure, which allows it to scavenge free radicals and reactive oxygen species effectively. FA's phenolic nucleus and carboxylic side chain form a resonance-stabilized phenoxy radical, rendering it highly effective in neutralizing oxidative stress [27]. Numerous studies have demonstrated the ability of FA to mitigate oxidative damage through various mechanisms. It has been shown to enhance the activity of endogenous antioxidant enzymes, such as superoxide dismutase, catalase, and glutathione peroxidase, thereby strengthening the body's natural defense against free radicals [28]. Moreover, FA can chelate metal ions, which can otherwise catalyze the formation of reactive oxygen species through Fenton reactions [29].

Anti-inflammatory Activity

FA exhibits anti-inflammatory activity by modulating key signaling pathways and molecular targets involved in inflammation. It inhibits the production and release of pro-inflammatory cytokines, such as TNF-α, IL-1β, and IL-6, which are critical mediators in the inflammatory response [30]. FA also downregulates the expression of enzymes like COX-2 and iNOS that are involved in the synthesis of pro-inflammatory molecules [31]. Furthermore, it suppresses the activation of nuclear factor-kappa B (NF-κB), a transcription factor regulating various inflammatory gene expression [32]. By attenuating oxidative stress, FA indirectly reduces inflammation, as oxidative stress is a known promoter of inflammatory pathways [33].

Antimicrobial Activity

FA exhibits promising antimicrobial activity against a broad spectrum of bacteria. It disrupts the bacterial cell membrane, a critical barrier protecting the cell. This disruption can lead to the leakage of essential cellular components and, ultimately, cell death. FA may also interfere with bacterial enzymes and protein synthesis, hindering their growth and reproduction. Additionally, it can interfere with biofilm formation, which is created by the sticky communities bacteria create to shield themselves from antimicrobials and the immune system. While the exact mechanisms may vary depending on the bacteria, FA’s multi-targeted approach makes it a potential weapon in the fight against microbial infections [34-36].

Cardioprotective Activity

FA exhibits cardioprotective activity by reducing oxidative stress, which is a key factor in cardiovascular diseases. It enhances the activity of antioxidant enzymes such as superoxide dismutase (SOD) and glutathione peroxidase (GPx), which neutralize harmful free radicals in the heart tissue. FA also improves endothelial function by increasing the bioavailability of nitric oxide (NO), a molecule crucial for vasodilation and maintaining blood vessel health [37]. Additionally, it inhibits the oxidation of low-density lipoprotein, preventing the formation of atherosclerotic plaques that can lead to heart attacks and strokes. FA reduces inflammation by downregulating pro-inflammatory cytokines and enzymes, further protecting the cardiovascular system. Its ability to modulate lipid metabolism helps lower cholesterol levels, thus contributing to overall heart health [38,39].

Anticancer Activity

FA exhibits anticancer activity by inducing apoptosis, or programmed cell death, in cancer cells, thereby inhibiting their proliferation. It modulates key signaling pathways, such as the p53 and NF-κB pathways, which are involved in cell survival and apoptosis [40]. FA also arrests the cell cycle in cancer cells, preventing their division and growth. It inhibits angiogenesis and the formation of new blood vessels, which is crucial for tumor growth and metastasis. Additionally, FA reduces oxidative stress and inflammation, which are linked to cancer development [41]. Its antioxidant properties protect normal cells from DNA damage, reducing the risk of mutations that can lead to cancer. FA enhances the efficacy of chemotherapeutic agents and reduces their side effects by protecting normal tissues [42].

Neuroprotective Activity

FA exhibits neuroprotective activity by reducing oxidative stress, which is a significant factor because oxidative damage is a major contributor to neuronal death and the progression of neurodegenerative diseases such as Alzheimer's, Parkinson's, and Huntington's. By scavenging free radicals and upregulating antioxidant enzymes such as SOD and catalase, FA provides a robust defense against oxidative damage. In addition to its antioxidant properties, FA's anti-inflammatory effects are significant, as chronic neuroinflammation exacerbates neuronal damage in neurodegenerative conditions. By downregulating pro-inflammatory cytokines such as TNF-α and IL-1β and inhibiting microglial activation, FA reduces the inflammatory environment contributing to neuronal injury [43,44]. Moreover, FA's ability to activate the Nrf2 signaling pathway is particularly important because Nrf2 is a key regulator of cellular defense mechanisms, including the expression of detoxifying and antioxidant genes [45]. This activation not only promotes neuronal survival but also enhances the brain's resilience to stress. Furthermore, FA's inhibition of amyloid-beta aggregation and tau phosphorylation addresses critical pathological features of Alzheimer's disease, potentially slowing disease progression and preserving cognitive function. By preventing the formation of neurofibrillary tangles and amyloid plaques, FA helps maintain neuronal integrity. Moreover, FA's enhancement of synaptic plasticity and neurotransmission underscores its role in supporting cognitive function and memory, making it a promising candidate for improving the quality of life in patients with neurodegenerative diseases [46,47].

Anti-aging Activity

FA exhibits anti-aging activity by neutralizing free radicals, thereby reducing oxidative stress, which is a major contributor to the aging process. It regenerates their active forms by enhancing the stability and effectiveness of other antioxidants, such as vitamins C and E. FA inhibits the degradation of collagen and elastin, crucial proteins that uphold skin elasticity and firmness. Additionally, it decreases the production of advanced glycation end products, contributing to skin rigidity and diminished elasticity. Additionally, FA modulates signaling pathways involved in cellular senescence, delaying the onset of aging-related cell changes. It improves skin hydration and texture, thereby enhancing overall skin appearance and reducing the appearance of wrinkles and fine lines [48-50].

Challenges associated with the conventional delivery of FA

The conventional delivery of FA faces several challenges that limit its effectiveness and therapeutic potential. These include poor bioavailability due to its low solubility in water and limited absorption in the gastrointestinal tract. FA undergoes rapid metabolism in the liver and is quickly eliminated from the body, reducing its systemic bioavailability and therapeutic efficacy [51,52]. Additionally, it is prone to degradation when exposed to light, heat, and oxygen, leading to a loss of potency over time [53]. The short biological half-life of FA necessitates frequent dosing to maintain therapeutic levels, which can be inconvenient and reduce patient compliance. Conventional delivery methods may not effectively target specific tissues or cells, limiting the potential for FA to exert its therapeutic effects in targeted areas of the body. It can also be degraded in the stomach's acidic environment and by enzymes in the gastrointestinal tract, further reducing its bioavailability. First-pass metabolism in the liver significantly reduces the effective dose of FA before it reaches systemic circulation. Additionally, FA has poor permeability across biological membranes, hindering its ability to reach target sites within the body. There can be significant variability in the absorption of FA due to factors such as diet, health status, and individual differences in metabolism, leading to inconsistent therapeutic outcomes [54,55]. Lastly, the non-specific distribution of FA after absorption means it is dispersed throughout the body rather than concentrated at the target site, reducing its efficacy and potentially causing off-target effects. These challenges highlight the need for novel drug delivery systems (i.e., NPs) to improve FA bioavailability, stability, and targeted delivery to enhance its therapeutic potential.

Novel drug delivery systems for FA

**Figure 2:**
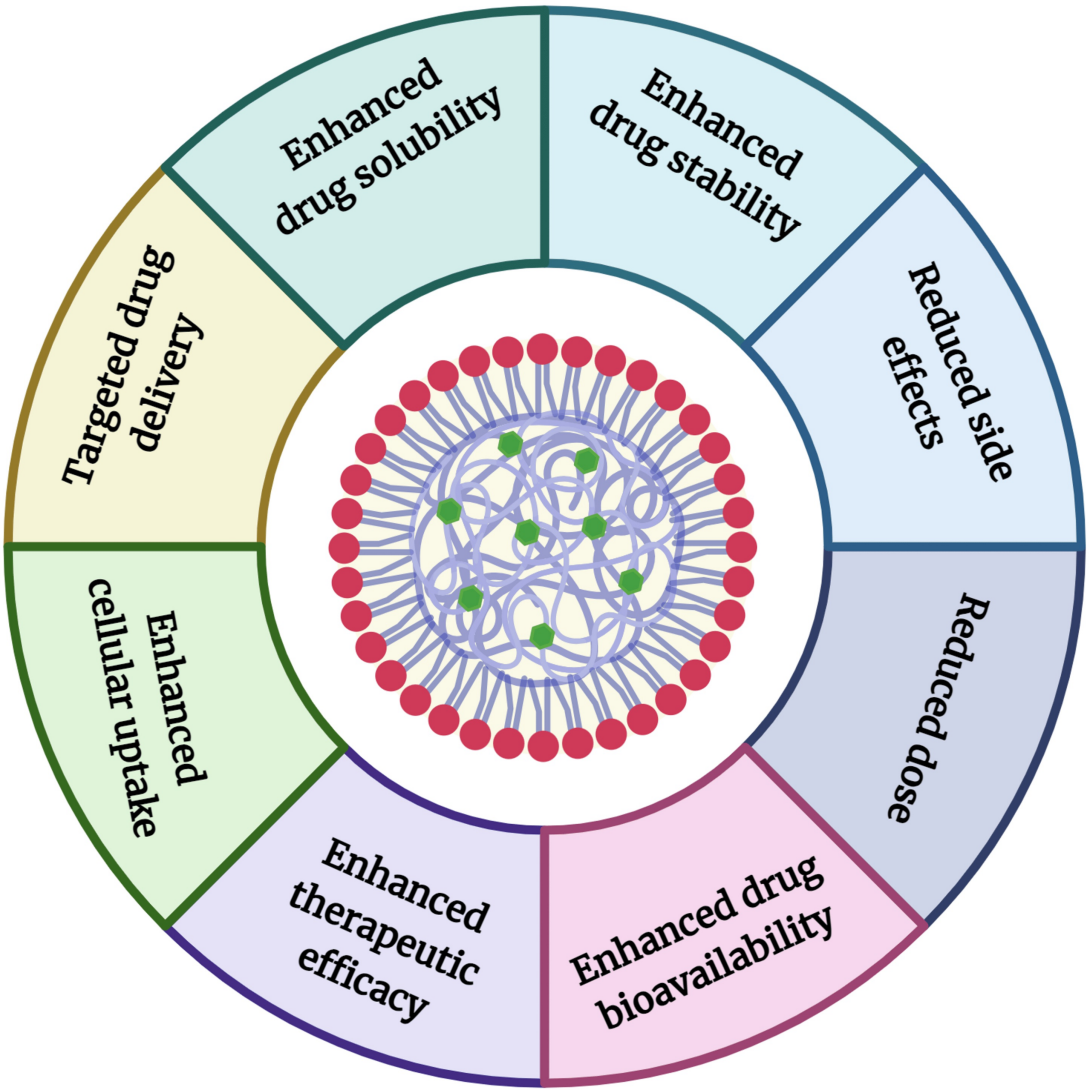
Diagrammatic representation of nanoparticles with their unique advantages. Image credit: Md. Rizwanullah

A novel drug delivery system (NDDS) refers to advanced methods and technologies designed to deliver pharmaceutical compounds in a controlled manner to achieve better therapeutic outcomes. NPs are the class of NDDS, defined as tiny particles ranging in size from 1 to 100 nm. They are used in various fields, including medicine, due to their unique properties at the nanoscale. In drug delivery, NPs can encapsulate therapeutic agents, protecting them from degradation and enhancing their stability, bioavailability, and targeted delivery to specific tissues or cells [56,57]. Their small size allows them to penetrate biological barriers and deliver drugs directly to the site of action, potentially reducing side effects and improving therapeutic outcomes. NPs can be made from various materials, such as lipids and polymers, each offering distinct advantages for different applications [58,59]. The diagrammatic representation of NPs with their unique advantages is represented in Figure 2. The various oral, intravenous, topical/transdermal, ocular, intranasal, and inhalation routes offer distinct advantages and open new avenues for the clinical application of FA in treating a wide range of diseases. The therapeutic delivery of FA with NPs by different routes is discussed below.

Oral Delivery

Oral delivery is one of the most convenient and widely accepted routes for drug administration due to its non-invasive nature, ease of administration, and patient compliance [60]. However, many drugs are lipophilic, leading to poor water solubility. Additionally, a significant number of these drugs are substrates for P-glycoprotein (P-gp) or the cytochrome P450 enzyme, or both, resulting in reduced oral bioavailability and therapeutic effectiveness due to expulsion or pre-systemic metabolism. Moreover, the harsh conditions within the gastrointestinal tract further hinder successful drug delivery through the oral route [61]. FA shows poor bioavailability primarily due to its low water solubility, which limits its dissolution and subsequent absorption in the gastrointestinal tract. Additionally, FA undergoes extensive first-pass metabolism in the liver, where it is rapidly conjugated through processes such as glucuronidation and sulfation, resulting in the formation of less active metabolites and a short half-life. The instability of FA in the gastrointestinal environment, particularly under acidic conditions and in the presence of digestive enzymes, leads to its degradation before it can be effectively absorbed [62].

NP-based delivery systems offer a promising solution to overcome these limitations and enhance the therapeutic efficacy of lipophilic drugs/phytochemicals. NP-based systems represent the ability to protect the drug from degradation in the harsh gastrointestinal environment, show controlled release characteristics, and significantly enhance the absorption from the intestine due to the high surface-to-volume ratio [63]. Therapeutic delivery of phytochemicals significantly reduces the dose and dosing frequency and the dose-related adverse effects [64,65]. Further, developing mucoadhesive NPs can also be an efficient approach. Mucoadhesive NPs can enhance the oral delivery of drugs/phytochemicals by adhering to the mucosal lining of the gastrointestinal tract, thereby increasing the residence time and allowing for prolonged drug absorption. This adhesion helps protect drugs/phytochemicals from the harsh gastric environment and enzymatic degradation. The improved retention at the absorption site enhances the bioavailability and efficacy of drugs/phytochemicals [66].

Parenteral Delivery

Parenteral drug delivery refers to the administration of drugs by injection or infusion directly into the body. This method is often used for poorly absorbed or unstable drugs/phytochemicals in the gastrointestinal tract [67]. The advantages of parenteral administration include rapid onset of action, precise control over drug dosing and blood levels, suitability for patients who cannot take oral medications, the ability to administer large volumes of fluids or medications, and targeted delivery to specific tissues or organs. It is also essential for emergency treatments and critical care situations [68]. However, parenteral drug delivery presents several challenges. It is invasive, requiring needles and injections, which can cause pain and increase the risk of infection. Strict aseptic techniques are necessary to prevent contamination, leading to higher costs due to sterile preparations and specialized equipment. Patient compliance can be difficult, especially for self-administration [69]. There are concerns about formulation stability and compatibility with body tissues, limited shelf life requiring careful storage, precise dosing, and monitoring to avoid errors, complex manufacturing processes, and a higher risk of immediate and severe adverse reactions [70]. Due to the FA's poor water solubility characteristics, the development of a parenteral formulation of FA is complicated. Further, FA is also prone to rapid degradation in the bloodstream, particularly through oxidation, which reduces its stability and therapeutic effectiveness. Its rapid clearance from the circulation due to metabolism by liver enzymes leads to a short half-life, requiring frequent dosing to maintain therapeutic concentration. The lack of targeted delivery in systemic circulation can result in off-target effects and reduced efficacy, as FA is distributed non-specifically throughout the body [71].

NPs enhance the stability of encapsulated drugs by protecting them from degradation and improving their solubility. They provide a controlled release of drugs or phytochemicals over an extended period, thereby increasing the plasma half-life. NPs can be functionalized with targeting ligands for site-specific and targeted drug delivery. This targeted delivery improves drug efficacy and reduces side effects. Additionally, NPs can cross biological barriers more effectively, enable combination therapies by carrying multiple agents, and allow for the delivery of drugs that are otherwise unstable or poorly soluble. They also enhance patient compliance through less frequent dosing and provide a platform for theranostic applications, combining therapeutic and diagnostic functions in a single system [72-74].

Topical/Transdermal Delivery

Topical/transdermal drug delivery involves administering drugs through the skin to achieve systemic effects. This method utilizes specially designed patches or formulations that release the drug at a controlled rate, allowing it to penetrate the skin and enter the bloodstream [75,76]. The advantages of transdermal drug delivery include avoiding the gastrointestinal tract and first-pass metabolism by the liver, which can degrade some drugs and reduce their efficacy. It provides a non-invasive and convenient alternative to oral and injectable routes, enhancing patient compliance. However, it is limited by the drug's molecular size and lipophilicity, as only small lipophilic molecules can effectively penetrate the skin barrier [77]. FA shows poor skin permeability due to its hydrophilic nature, which limits its ability to penetrate the stratum corneum. FA's low stability in the presence of light, oxygen, and heat can lead to rapid degradation, reducing its efficacy when applied topically/transdermally. Additionally, FA may undergo oxidation on the skin surface, diminishing its antioxidant properties [78].

NPs play a significant role in improving transdermal drug/phytochemical delivery by enhancing the penetration and efficacy of drugs. NPs can penetrate the stratum corneum, the outermost layer of the skin, more effectively than conventional formulations. Their small size allows them to navigate through the skin's lipid matrix and reach deeper layers [79]. NPs protect encapsulated drugs from degradation by environmental factors, such as light and air, and from enzymatic activity within the skin, thereby enhancing drug stability. NPs can improve the solubility of poorly soluble drugs, ensuring that a sufficient amount of the drug is available for absorption through the skin [80]. NPs can be functionalized with targeting ligands to direct the drug to specific cells or tissues, thereby enhancing therapeutic efficacy and reducing side effects [81]. Overall, the use of NPs in transdermal drug delivery systems can offer a more convenient and less invasive alternative to oral or injectable routes, improving patient compliance.

Pulmonary Delivery

Pulmonary drug delivery involves administering drugs directly into the lungs to treat respiratory diseases or to achieve systemic effects. This method utilizes inhalers, nebulizers, or dry powder inhalers to deliver drugs in the form of aerosols, mists, or dry powders [82,83]. Pulmonary drug delivery offers the advantage of a rapid onset of action, owing to the lungs' large surface area and extensive blood supply, allowing quick absorption into the bloodstream. It provides targeted delivery to the respiratory system. Pulmonary delivery also avoids first-pass metabolism by the liver, improving the bioavailability of drugs that are otherwise degraded in the GIT [84]. It allows for non-invasive administration, enhancing patient compliance, especially for those who have difficulty swallowing or require frequent dosing. However, challenges include ensuring uniform drug distribution within the lungs, avoiding drug deposition in the mouth or throat, and the need for precise dosing [85].

The development of the conventional inhalable formulation of FA is complicated due to its poor solubility in aqueous media. Further, FA's instability when exposed to air, light, and heat can lead to degradation during formulation, storage, or delivery, reducing its therapeutic efficacy. Additionally, the rapid clearance of FA from the lungs, primarily through mucociliary mechanisms and enzymatic metabolism, limits its residence time in the pulmonary system, necessitating frequent dosing to maintain therapeutic levels [86].

NPs can circumvent the challenges associated with conventional pulmonary drug delivery. NPs improve the solubility of poorly water-soluble drugs, ensuring effective delivery to the lungs. NPs can be designed to enable controlled and sustained drug release, maintaining therapeutic levels over extended periods and reducing the need for frequent administration [87]. NPs can be functionalized with ligands to target specific cells or tissues within the lungs, enhancing drug efficacy and minimizing side effects. NPs increase the absorption of drugs by facilitating their transport across the lung epithelium into the bloodstream. By providing localized delivery to the lungs, NPs minimize systemic exposure and significantly decrease the risk of adverse effects associated with the drug [88,89].

Intranasal Delivery

Intranasal delivery refers to the administration of drugs through the nasal cavity. This method takes advantage of the rich blood supply and the large surface area of the nasal mucosa, which allows for rapid absorption of medications directly into the bloodstream [90]. It bypasses the digestive system and pre-systemic metabolism in the liver, leading to a faster onset of action. This route is particularly useful for drugs that require quick effects, especially diseases related to the central nervous system (CNS). It can also provide a non-invasive alternative for patients with difficulty swallowing pills or needing immediate relief [91,92]. However, variability in absorption due to nasal congestion or other nasal conditions and limited area for absorption limits its application. Further, different biological barriers also limit the application of this route [93]. The poor aqueous solubility characteristics of FA significantly limit its absorption across the nasal mucosa. FA's rapid metabolism by nasal enzymes, such as cytochrome P450s, can lead to its degradation before it reaches the systemic circulation or CNS, reducing its bioavailability and therapeutic efficacy. The mucociliary clearance in the nasal cavity quickly removes substances, necessitating frequent administration to maintain effective drug levels. FA's instability can further reduce its potency when exposed to air, light, and nasal secretions. Additionally, the limited surface area of the nasal cavity and the blood-brain barrier present significant obstacles to effective delivery, particularly if targeting the CNS. The potential for local irritation or sensitization of the nasal mucosa at higher concentrations also poses a risk, complicating formulation and dosing strategies [94].

The intranasal delivery of drug/phytochemicals with NPs can be an alternative approach to overcome the above-discussed challenges. NPs significantly improve intranasal drug delivery by improving drug stability, bioavailability, and targeting. NPs protect drugs from degradation and provide controlled release, ensuring a sustained therapeutic effect. NPs facilitate the transport of drugs across the nasal mucosa and can be engineered to overcome the barriers of nasal enzymes and mucociliary clearance [95,96]. NPs enhance drug absorption and penetration by interacting with the nasal epithelial cells and opening tight junctions. Their small size and surface properties can be tailored to optimize drug delivery and patient compliance [97].

Ocular Delivery

Ocular delivery refers to the administration of drugs or therapeutic agents directly into the eye to treat ocular diseases, ensuring targeted and effective treatment while minimizing systemic side effects [98]. This method is specifically designed to deliver medication to the eye, where it is needed most, thereby enhancing the therapeutic effects and reducing the potential for side effects that might occur if the drug were distributed throughout the body. The advantages of ocular drug delivery include targeted therapy directly to the site of action, which enhances drug efficacy and reduces systemic side effects, and it provides a non-invasive, patient-friendly method of administration [99]. The limitations include the eye's natural barriers, such as tear production, blinking, and drainage that reduce drug bioavailability, potential irritation or discomfort to the patient, and the challenge of achieving and maintaining therapeutic drug concentrations in the target tissues [100]. The development of the ocular formulation of FA is hindered by its poor solubility in aqueous solutions. FA's rapid degradation when exposed to light, air, and ocular fluids reduces its stability and therapeutic efficacy. The presence of ocular barriers, such as the corneal epithelium and tear film, limits the penetration of FA into deeper ocular tissues, resulting in low bioavailability. The rapid clearance of FA from the ocular surface due to lacrimation and blinking necessitates frequent administration to maintain effective drug concentrations. Additionally, FA's potential to cause irritation or hypersensitivity reactions in the delicate ocular tissues, especially at higher concentrations, poses a risk in ocular formulations [101].

The fabrication of NPs for ocular drug/phytochemical delivery can address the associated challenges. NPs for ocular drug delivery offer enhanced drug bioavailability and therapeutic efficacy by overcoming the eye’s natural barriers. NPs provide sustained and controlled drug release, reducing the need for frequent dosing and improving patient compliance. These NPs can be designed to target specific ocular tissues, ensuring precise delivery and minimizing systemic side effects. Additionally, they protect drugs from enzymatic degradation and increase their residence time on the ocular surface, enhancing overall treatment effectiveness [102,103].

Different NPs for therapeutic delivery of FA

NP-based delivery systems offer a promising approach to overcoming the limitations of FA, enhancing its therapeutic potential across various applications. Different types of NPs for the therapeutic delivery of FA are discussed below, and a comparative analysis of different NPs is summarized in Table 1.

**Table 1:** Summary of the advantages and limitations of different nanoparticle-based systems.

Nanoparticles	Advantages	Limitations
Polymeric nanoparticles	▪High stability and controlled drug release ▪Ability to encapsulate a wide range of drugs ▪Biocompatibility and potential for targeted delivery	▪Complex and costly synthesis ▪Possible cytotoxicity of certain polymers ▪Limited drug loading capacity for some drugs
Polymeric micelles	▪Enhanced solubility of poorly soluble drugs ▪Targeted delivery through surface modification ▪Improved pharmacokinetics and biodistribution	▪Low physical stability ▪Potential premature drug release ▪Limited drug loading capacity
Liposomes	▪Biocompatibility and biodegradability ▪Ability to encapsulate both hydrophilic and lipophilic drugs ▪Reduced toxicity of encapsulated drugs	▪High production cost ▪Limited stability (prone to leakage) ▪Short circulation time without modification
Nanoemulsions	▪High solubilization capacity for hydrophobic drugs ▪Ease of production and scale-up ▪Improved drug absorption and bioavailability	▪Stability issues (creaming, flocculation, coalescence) ▪Limited drug loading capacity ▪Possible irritation at high surfactant concentrations
Solid lipid nanoparticles	▪High stability and controlled release ▪Protection of labile drugs from degradation ▪Biocompatibility	▪Limited drug loading capacity ▪Potential drug expulsion during storage ▪Complexity in large-scale production
Nanostructured lipid carriers	▪Higher drug loading capacity compared to SLNs ▪Enhanced stability and controlled release ▪Reduced drug expulsion during storage	▪Complexity in formulation design ▪Potential cytotoxicity due to the use of organic solvent ▪Higher production costs

Polymeric NPs

Polymeric NPs, composed of natural or synthetic polymers, are extensively utilized in drug delivery systems due to their biocompatibility and biodegradability. These NPs can provide controlled and sustained release of encapsulated drug/phytochemical. By engineering specific properties such as size, surface charge, and functionalization with targeting ligands, their delivery efficiency and efficacy can be significantly enhanced [104]. Additionally, polymeric NPs can traverse different biological barriers to targeted specific sites within the body, encapsulate a wide array of therapeutic substances, and release them efficiently in response to internal and external stimuli [105]. To investigate the potential of these NPs, Arınmış et al. fabricated FA-encapsulated poly lactic-co-glycolic acid (PLGA)-based NPs for enhanced anti-Alzheimer activity [106]. The developed FA-PLGA-NPs revealed excellent pharmaceutical attributes such as particle size, zeta potential (ZP), and entrapment efficiency (EE). The nanocarrier revealed a sustained and prolonged release of FA up to 24 hours and represents excellent stability in the gastrointestinal milieu. The developed NPs represented excellent antioxidant potential by strong DPPH free radical scavenging. Further, the developed NPs showed excellent anti-Alzheimer activity with strong anti-acetylcholinesterase activity. In another investigation, Panwar et al. fabricated FA-encapsulated chitosan NPs for improved anti-diabetic activity [107]. The pharmacokinetic experiment in Wistar albino rats showed that the developed NPs rapidly absorbed and showed comparatively higher oral bioavailability than pure FA. Further, the developed NPs represented significantly better anti-diabetic activity by decreasing blood glucose levels and improving the lipid profiles in streptozotocin-induced diabetic rats.

Polymeric Micelles

Since the start of the 21st century, polymeric micelles have become a highly promising platform for the delivery of therapeutic compounds. Polymeric micelles are nanosized colloidal particles formed by the self-assembly of amphiphilic block copolymers in aqueous environments. They have a core-shell structure, a lipophilic core that can entrap lipophilic drugs and a hydrophilic shell that stabilizes the micelle in the aqueous medium. This unique architecture makes polymeric micelles particularly useful for drug delivery applications [108]. In preclinical animal models, polymeric micelles have demonstrated excellent pharmacokinetic profiles, higher efficacy, and better safety. Several formulations of polymeric micelles have advanced to the clinical stage and are either undergoing clinical trials or have been approved for human use [109]. In a study, Grimaudo et al. fabricated FA-encapsulated polymeric micelles-based nanogel for improved ocular delivery [110]. The developed nanocarrier revealed excellent pharmaceutical attributes. The nanocarriers represented excellent biocompatibility and showed sustained release profiles. In addition, the nanocarriers represented better in vitro antioxidant activity and wound healing activity. Further, nanocarriers represented much higher ex vivo accumulation of FA in both healthy and damaged corneas.

Liposomes

Liposomes are spherical vesicular systems composed of lipids featuring a bilayer structure where hydrophilic layers surround a lipophilic core [111]. Liposomes can encapsulate both hydrophilic and lipophilic drugs, making them versatile carriers for drug delivery. Due to their excellent biocompatibility and biodegradability characteristics, liposomes are considered safe for use in medical applications. These properties are crucial because they reduce the risk of toxicity and other adverse effects associated with drug delivery systems. Liposomes can be engineered for targeted drug delivery, allowing for increased drug concentration at specific sites while minimizing systemic exposure and toxicity. They also enhance the pharmacokinetics of drugs by prolonging their circulation time, improving bioavailability, and enabling controlled or sustained release [112,113]. Further, they enhance the therapeutic outcomes of encapsulated drug/phytochemicals by providing stability, overcoming biological and physiological barriers, enhancing drug biodistribution, and targeted delivery to the target site [114]. In this context, Ara et al. fabricated FA-encapsulated liposomes to obtain better therapeutic efficacy in managing liver damage [115]. The developed FA-liposomes represented potent scavenging efficacy against hydroxyl radicals and prevented CCl4-mediated cytotoxicity in human hepatocarcinoma cells. Consequently, FA-liposomes markedly decrease alanine aminotransferase and aspartate aminotransferase levels in a rat model of liver injury after intravenous administration. Further, the histological section of the liver confirmed the hepatoprotective effects of the developed FA-liposomes.

Nanoemulsion

Nanoemulsions are emulsions stabilized kinetically, with droplets sized in the nanometer range. These tiny droplets provide confined spaces where polymerization or precipitation reactions can occur, facilitating the formation of particles and capsules that serve as nanocarriers for biomedical uses. Nanoemulsions are highly adaptable systems used to encapsulate components dissolved within their dispersed phase. Direct systems, such as oil-in-water nanoemulsions, are effective for encapsulating lipophilic components. Conversely, inverse systems facilitate the encapsulation of hydrophilic cargo with ease [116]. Nanoemulsions have the potential to address various challenges in drug formulation. Encapsulating poorly water-soluble drugs in suitable nanoemulsions enhances their wettability and solubility. As a result, this improves the drugs' pharmacokinetics and pharmacodynamics through different routes of administration [117]. In a study, Harwansh et al. fabricated FA-loaded nanoemulsion for topical application for better skin protection activity [118]. The developed FA-loaded nanoemulsion showed a sustained release profile, higher skin permeability potential, and better ultraviolet A (UVA) protection than the conventional FA suspension. Better results were achieved due to enhanced solubility and permeability from the developed nanoemulsion, improving the levels of skin marker enzymes and providing protection against UVA-induced oxidative stress.

Solid Lipid NPs

Solid lipid NPs (SLNs) comprise a solid lipid core, mainly containing glycerides, fatty acids, sterols, or waxy molecules that are surface-stabilized by emulsifiers. SLNs can solubilize and retain lipophilic molecules in their core, allowing for drug delivery of compounds that would be poorly stabilized by other vectors, such as liposomes or oil-in-water nanoemulsions [119]. Key benefits include adjustable drug release, improved bioavailability, protection of chemically unstable compounds such as retinol from degradation, cost-effective excipients, and a wide range of applications [120]. In a study, Saini et al. prepared FA-loaded chitosan-coated SLNs for managing Alzheimer's disease [121]. The developed SLNs represented markedly improved ex vivo mucoadhesion and permeation of the drug compared to the pure FA. In vivo studies revealed substantial enhancement in cognitive ability via the reduction in escape latency time after intranasal administration of the developed SLNs compared to the pure FA. Further, the developed SLNs exhibited notable enhancement in different biochemical parameters and body weight gain in rats. Overall, the therapeutic delivery of FA by encapsulation into the chitosan-coated SLNs can be a potentially better approach for managing Alzheimer's disease.

Nanostructured Lipid Carriers

NLCs contain a lipidic-liquid interface, usually consisting of oils like oleic acid, and typically embedded within a solid core such as an SLN [122]. NLCs can further enhance the solubility and stability of certain compounds that are not well solubilized in the solid core of SLNs, making them a suitable delivery vector for the potential co-encapsulation of drugs in either the oil phase, the solid phase, or both depending on the matrix mixture and drug properties [123]. At room temperature, they possess a solid matrix and are regarded as better than other traditional lipid-based nanocarriers. This superiority is attributed to their enhanced physical stability, higher drug loading capacity, and improved biocompatibility [124]. In this context, Zhang et al. fabricated trans-FA-encapsulated NLCs for improved oral bioavailability [125]. The developed NLCs represented excellent pharmaceutical attributes and better storage stability. The fabricated NLCs exhibited a biphasic release profile with fast release at first and then a sustained release for a longer period. The pharmacokinetic study was conducted using Sprague-Dawley (SD) rats. After oral administration, the developed NLCs represented more than five times in comparison with the pure FA suspension.

Future perspectives

While nanotechnology offers promising solutions for FA delivery, there are still challenges to overcome and exciting future directions to explore. Tailoring NPs for specific disease targets and desired release profiles remains crucial. Controlled release properties are essential to ensure sustained drug delivery and minimize the need for frequent dosing. Additionally, optimizing particle size, surface charge, and targeting ligands is necessary for efficient cellular uptake and targeted delivery to diseased tissues [126,127]. Extensive in vivo investigations are required to evaluate the efficacy and safety of FA-loaded NPs. These studies should assess pharmacokinetics, biodistribution, and potential side effects. Developing cost-effective and scalable production methods for FA-loaded NPs is critical for wider clinical application. Large-scale production techniques need to be established to ensure the affordability and accessibility of these therapies. Exploring the potential of combining FA-loaded NPs with other therapeutic agents for synergistic effects holds promise. For example, combining FA with chemotherapeutic drugs for cancer treatment could enhance efficacy while reducing side effects [128,129]. Research into alternative delivery systems beyond NPs is also important. Stimuli-responsive polymers that release FA in response to specific triggers such as pH or light could offer targeted and controlled drug delivery. Additionally, investigating the potential of natural biopolymers for NPs development could provide more sustainable and potentially less toxic options [130,131]. By addressing these challenges and pursuing these future directions, FA-loaded NPs have the potential to revolutionize the treatment of various diseases. As research progresses, we can expect to see these innovative drug delivery systems translated into clinical practice, offering patients new and improved therapeutic options.

## Conclusions

FA has emerged as a promising therapeutic agent with a broad spectrum of pharmacological activities, including antioxidant, anti-inflammatory, and anticancer properties. However, its clinical translation is hampered by limitations such as poor water solubility, low bioavailability, and rapid metabolism. Nanotechnology offers a transformative approach to addressing these challenges by utilizing NPs for FA delivery. NPs can enhance solubility, stability, and bioavailability, ultimately improving the therapeutic efficacy of FA. This review has comprehensively discussed the therapeutic potential of FA, the limitations associated with its conventional delivery, and the possibilities offered by novel drug delivery systems, particularly NPs. Polymeric and lipid-based NPs have shown promise in delivering FA for managing various diseases. While nanotechnology presents a significant step forward, future research should focus on optimizing NPs design for targeted delivery. Exploring combination therapies with other therapeutic agents and investigating alternative delivery systems such as stimuli-responsive polymers hold promise for further advancements.
